# Regard and protect ground‐nesting pollinators as part of soil biodiversity

**DOI:** 10.1002/eap.2564

**Published:** 2022-03-17

**Authors:** Stefanie Christmann

**Affiliations:** ^1^ International Center for Agricultural Research in Dry Areas (ICARDA) Rabat Morocco

**Keywords:** CBD, FAO, ground‐nesting pollinators, habitat, mycorrhizal fungi, pesticides, soil biodiversity

## Abstract

While the Convention on Biological Diversity employs a habitat‐oriented definition of soil biodiversity including all kinds of species living in soil, the Food and Agriculture Organization, since 2002 assigned to safeguard soil biodiversity, excludes them by focusing on species directly providing four ecosystem services contributing to soil quality and functions: nutrient cycling, regulation of water flow and storage, soil structure maintenance and erosion control, and carbon storage and regulation of atmospheric composition. Many solitary wasps and 70% of wild bees nest below ground and require protection during this long and crucial period of their lifecycle. Recent research has demonstrated the extent of threats to which ground‐nesting pollinators are exposed, for example, chemicals and deep tillage. Ground‐nesting pollinators change soil texture directly by digging cavities, but more importantly by their indirect contribution to soil quality and functions: 87% of all flowering plants require pollinators. Without pollinators, soil would lose all ecosystem services provided by these flowering plants, for example, litter, shade, roots for habitats, and erosion control. Above‐ and belowground biota are in constant interaction. Therefore, and in line with the Convention's definition, the key stakeholder, the Food and Agriculture Organization should protect ground‐nesting pollinators explicitly within soil biodiversity conservation.

## INTRODUCTION

Eighty‐seven percent of flowering plants (Ollerton et al., 2011) and 75% of the most important food crops (Klein et al., 2007) depend on pollinators. Wild bees are the most important group of pollinators, but globally they are in decline (Zattara & Aizen, 2021). About 70% of recognized wild bees nest in the ground, also many solitary wasps and some syrphids (Antoine & Forrest, 2021; Cope et al., 2019; Hopwood et al., 2021). The value of pollinators for soil biodiversity and soil functions has never been assessed.

## MAIN STAKEHOLDERS DO NOT BUILD ON THE CBD DEFINITION OF SOIL BIODIVERSITY

The Convention on Biological Diversity (CBD) defines soil biodiversity as “the variety of life below ground, from genes and species to the communities they form, as well as the ecological complexes to which they contribute and to which they belong, from soil microhabitats to landscapes” (CBD, 2020). The habitat‐oriented definition allows protection of ground‐nesting pollinators as part of soil biodiversity.

CBD charged The Food and Agriculture Organization (FAO) with global conservation of soil biodiversity and with the International Pollinator Initiative (CBD, 2002, COP 6, decision V/5, section II, https://www.cbd.int/decision/cop/?id=7179). However, the *FAO Voluntary Guidelines for Sustainable Soil Management* (FAO, 2017) and the full report *State of Knowledge on Soil Biodiversity* (FAO, 2020) do not include ground‐nesting pollinators as part of soil biodiversity. Though the 616‐page report (FAO, 2020) directly refers to the CBD definition in the beginning, the following sub‐chapter on soil communities excludes pollinators and lists only species directly contributing to soil quality and functions. Pollinator decline is mentioned superficially four times in reports on certain countries without any reference to specific threats to ground‐nesting pollinators (FAO, 2020). The Global Soil Partnership (GSP), founded by FAO in 2012, narrows the focus: “Soil depends on the presence of a vast community of living organisms to remain healthy and fertile: these organisms make up soil biodiversity” or “Soil biodiversity plays a vital role in the soil ecosystem as soil organisms are responsible for nutrient cycling, regulating the dynamics of soil organic matter, soil carbon sequestration and greenhouse gas emissions, allowing soils to function properly” (http://www.fao.org/global-soil-partnership/areas-of-work/soil-biodiversity/en/). Soil research focuses on organisms (moles, beetles, ants, termites, earthworms, millipedes, woodlice, tardigrades, mites, nematodes, fungi, bacteria, protozoans, etc.) directly providing four ecosystem services contributing to soil quality and functions: nutrient cycling, regulation of water flow and storage, soil structure maintenance (including detoxification of xenobiotics and pollutants) and erosion control, carbon storage and regulation of atmospheric composition (FAO, 2015, 2020). The 650‐page FAO report on the world's soil resources mentions that biodiversity above‐ground, particularly pollinators and natural enemies provide important ecosystem services and are threatened by pesticides but without any reference to specific threats to ground‐nesting pollinators (FAO, 2015).

The EU Thematic Strategy for Soil Protection (EU, 2006) mentions all “living organisms” of the “top layer of the earth's crust” but does not clarify which species are included. The EU Global Soil Atlas (Orgiazzi, Bardgett, et al., 2016) briefly acknowledges the importance of pollinators, seed eaters and seed dispersers for plant distribution but confines itself to species providing the above‐mentioned ecosystem services and soil functions directly. If some of these species contribute additionally to pollination, for instance ants and beetles, this is highlighted (Orgiazzi, Bardgett, et al., 2016). The authors mention that around 70% of recognized wild bees nest in soil and state that mining bees “in reasonable numbers […] will not harm your garden” (Orgiazzi, Bardgett, et al., 2016). Neither the soil‐related specific threats for ground‐nesting pollinators nor protection strategies are described. The European Soil Data Center (ESDAC; https://esdac.jrc.ec.europa.eu/themes/soil-biodiversity) also does not include ground‐nesting pollinators. As CBD charged FAO with both global pollinator protection and global conservation of soil biodiversity explicitly to promote synergies (CBD 2002, COP 6, decision V/5, section II, https://www.cbd.int/decision/cop/?id=7179), it is striking that FAO did not take the lead to integrate ground‐nesting pollinators in soil protection.

Ground‐nesting pollinators should be explicitly included in concepts of soil biodiversity and respective protection guidelines and programs, because (1) soil is an essential habitat for ground‐nesting pollinators where they face specific threats, (2) indirectly, ground‐nesting pollinators contribute to soil biodiversity by (a) ensuring regeneration of flowering plants and thus sustain diverse habitats above and below ground, (b) enabling the adaptation of flowering plants to climate change through cross‐pollination that enhances genetic diversity (MEA, 2005), and (3) ground‐nesting pollinators directly contribute to soil structure by aerating the soil through microtillage (Cane, 2003). Carbon storage, a main soil function (FAO, 2020) highly depends on pollinator‐dependent flowering plants, the contributions of pollinators to climate‐change mitigation through soil are multifold but neither precisely assessed nor commonly recognized (Christmann, 2019).

## SOIL AS HABITAT: SPECIFIC THREATS TO GROUND‐NESTING POLLINATORS

Most wild pollinators work in a 50–2000 m radius around the nest (Kohler et al., 2008) with much smaller radii (150–600 m) identified for foraging trips of 16 solitary bees (Gathmann & Tscharntke, 2002). The lower the foraging radius, the higher the eventual exposure to pesticides, making solitary bees more exposed to pesticides than social bees (Uhl & Brühl, 2019).

Many solitary wasps nest in soil, wild bees nest either in soil (*Halictus*, *Lasioglossum*, *Andrena*, *Eucera*, *Anthophora*, *Amegilla* and *Melitta*) or in hollow stems or old wood (*Xylocopa*, *Megachile* and *Osmia*). In monocultural areas with large field sizes, field edges become rare (Feltham et al., 2015), and dead wood, hollow stems, and perennial aboveground biomass for nesting are even more scarce, thus disadvantaging cavity‐nesting pollinators (Brown et al., 2020; Everaars, 2012). They live closer to forest patches (Everaars, 2012). Long distances between nests and foraging plants reduce pollination services (Roulston & Goodell, 2011). As such, agriculture depends to a high extent on ground‐nesting pollinators that nest in the entire field area (Everaars, 2012; Sgolastra et al., 2018). Pollinators nesting in the soil of fields face three additional main threats in comparison to honeybees and cavity‐nesting pollinators: tillage, soil compaction by heavy machinery, and chemicals accumulating in soil.

Little is known about the impacts of tillage on the regeneration of solitary ground‐nesting pollinators (Kratschmer et al., 2018). They usually stay in the ground almost a full year, sometimes several years, emerging for some weeks or months to reproduce, pollinate, and lay eggs (Michener, 2007; Willis Chan et al., 2019). Some bees prefer tilled soil to build nests (Skidmore et al., 2019). However, as many ground‐nesting pollinators place their eggs up to 30 cm below surface (Roulston & Goodell, 2011), deep tillage (around 15–30 cm) can bury nests or destroy the tunnel (Hopwood et al., 2021; Roulston & Goodell, 2011). Tillage reduces offspring emergence and delays emergence of surviving offspring of ground‐nesting squash bees (Ullmann et al., 2016). No‐tillage farms hosted three times higher density of squash bees as farms employing tillage (Shuler et al., 2005).

Soil compacted by heavy machinery threatens ground‐nesting pollinators (Potts & Willmer, 2003). As burrowing in compacted soil is hampered even for earthworms (Beylich et al., 2010), heavy machinery might compact soil to an extent that pollinator larvae are smashed or cannot emerge successfully from their natal nests. Compacted soil has reduced macropore volume and thus different O_2_ and CO_2_ concentrations affecting soil organisms (Beylich et al., 2010). Effects of these different concentrations on hatching offspring and pollinator females digging to lay eggs have not yet been studied. In compacted soil it is possible that fungal diseases may impact non mobile stages (pupae) of ground‐nesting pollinators due to reduced soil water drainage. The impacts of different soil tillage regimes at different times on the reproduction of ground‐nesting pollinators should be assessed to enhance guidelines for less damaging soil cultivation.

Chemicals and neonicotinoid insecticides accumulate in soil, with up to 94% of the chemical load of neonicotinoids found in soil and water (Goulson, 2014). Female solitary ground‐nesting pollinators dig vertical and horizontal tunnels and cells in soil to lay their eggs, some species create deep and multibranched tunnel systems (Willis Chan et al., 2019). However, pesticide risk assessments are mostly conducted on honeybees (Uhl & Brühl, 2019), though honeybees are more mobile than wild pollinators and enjoy substantial support by beekeepers (hives, transport). To a lesser extent, pesticide risk assessments include bumblebees (Hatfield et al., 2021) that occupy existing mouse holes for their nests and cavity‐nesting species like *Osmia* (Rundlöf et al., 2015). Pesticide risk assessments rarely include the most exposed groups, ground‐nesting pollinators or aquatic syrphids (Anderson & Harmon‐Threatt, 2019; Goulson, 2014; Rundlöf et al., 2015; Sgolastra et al., 2018; Willis Chan et al., 2019; Willis Chan & Raine, 2021). Exposure to imidacloprid reduces the life expectancy of solitary bees (Anderson & Harmon‐Threatt, 2019). Hoary squash bees in *Curcubita* fields in Ontario exposed to imidacloprid build 85% fewer nests and their next generation is reduced by 89% (Willis Chan & Raine, 2021). The toxicity for larvae has not yet been tested (Willis Chan et al., 2019). Wild bees’ ability to reproduce may be reduced even in the year following exposure to neonicotinoids (Woodcock et al., 2017). Elado (a combination of clothianidin and the non‐systemic pyrethroid β‐cyfluthrin) decreases wild bee density, solitary bee nesting, growth of bumblebee colonies, and reproduction (Rundlöf et al., 2015).

Female solitary ground‐nesting pollinators are exposed to neonicotinoids with their entire bodies while digging. The time of contact exposure might be long, *Dasypoda visnaga* and *Dasypoda maura* dig up to 80 cm deep (El Abdouni et al., 2021). The exposure of different ground‐nesting pollinators can differ according to their body size, the size of nests, the month of nest‐building activities, preferred crops, farming practice, and precipitation, which may reduce the concentration of chemicals in soil. Research is needed on the interaction of different chemicals with each other in soils and how unintended side effects affect the total toxicity for ground‐nesting pollinators. This is even more urgent with respect to climate change not only, but notably in dry regions like North Africa, Near and Middle East, and Asia (pests are not washed away by rain, they expand regionally, regenerate faster, and drought‐stressed plants attract more pests) (Deutsch et al., 2018; Skendžić et al., 2021). Farmers can increase the diversity of pesticides used, their concentration, and the frequency of application. Also, the concentration in soil can increase due to higher evaporation. Research is needed on the duration of contact exposure of different female ground‐nesting pollinators in soil and how this impacts their health (Willis Chan & Raine, 2021).

The urgent need to protect ground‐nesting pollinators below ground using soil‐protection strategies is obvious. As pesticides and particularly neonicotinoids affect other species of soil biodiversity like earthworms (Pisa et al., 2014), ants (Schläppi et al., 2020) and even aboveground species like birds (Goulson, 2014) and bats (Hsiao et al., 2016), stronger collaboration of scientists and policymakers can be advantageous.

## MULTIFOLD INTERACTIONS BETWEEN SPATIALLY SEPARATED BIOTA

Various impacts of aboveground biodiversity on belowground biodiversity and soil (Porazinska et al., 2018; Wardle et al., 2004) and vice versa have been described (de Deyn et al., 2003), litter above ground impacts decomposer biota below ground (Ball et al., 2009), most plants interact with both mycorrhizal fungi and animal pollinators; changes in above‐ or belowground richness and abundance affect species and functions in the other habitat (Brody et al., 2021), absence of mycorrhizae can reduce diversity of plants and abundance of pollinators, while herbivores attacking roots and mycorrhizal colonization support flower visitation by pollinators (A'Bear et al., 2014). Symbiotic and pathogenic soil microbes impact the composition of vegetation of grasslands (Wardle et al., 2004). Climate‐change induced events (earlier snowmelt, drought) lead to reduced mutualistic interactions between plants, mycorrhizal fungi, and pollinators (Keeler et al., 2021). Floods and soil run‐off can move small soil organisms downstream, where they can alter below‐ and aboveground biota (Orgiazzi & Panagos, 2018).

The complexity of interactions between spatially separated sub‐communities and the impacts on food chains in fauna have been highlighted in principle (Goulson, 2014; Knight et al., 2005). Knight et al. (2005) analyze them using the example of fish feeding on dragonfly larvae, while adult dragonflies live on pollinators and other insects. Thus, the availability of fish in the pond has positive impacts on pollinator availability and the diversity of plants and plant reproductive success in the land surrounding the lake.

## IMPACTS OF POLLINATOR LOSS ON SOIL BIODIVERSITY, SOIL FERTILITY, AND EROSION PREVENTION

These examples highlight the need to take processes within landscapes into consideration. As 87% of all flowering plants depend on pollinators (Ollerton et al., 2011), all ecosystem services provided by those plants also depend on pollinators (Christmann, 2019). Loss of 87% of flowering plants would reduce and change plant diversity, and in consequence diversity of fauna and habitats (Christmann, 2019). Pollinator‐loss‐induced changes of vegetation and fauna would impact soil biodiversity and soil functions, water run‐off, recharge of aquifers, reduce filtering against pollution and increase exposure to sun (particularly in case of loss of trees), change ambient humidity and temperature above and below ground, nutrient cycling, root biomass and root diversity, carbon storage, etc. (Christmann, 2019), and the local environment for mycorrhizal fungi, earthworms, ants, dung beetles, and other soil biota could alter rapidly.

In particular, organizations working on soil biodiversity need an integrated view of landscapes as the ability of most soil organisms to move fast is limited while they are subject to changes in the landscape around. Soil bacteria and protists, for example, can move actively around 0.000001 m only, nematodes 0.01 m, and arbuscular mycorrhizal fungi 0.005 m per day (Orgiazzi, Bardgett, et al., 2016). Loss of pollinator‐dependent plants, litter and roots can have vast impacts on soil bacteria, mycorrhizal fungi, and other soil species, particularly on mutualistic soil organisms. Higher soil erosion directly affects soil organisms (Orgiazzi & Panagos, 2018). Pollinator loss in a region can cause interlinked degradation spirals reducing the options for restoration (Christmann, 2019). However, publications on threats to soil diversity do not even mention pollinator loss but declare wind and water as highest threats (FAO, 2015, 2017; Orgiazzi, Panagos, et al., 2016), though high plant diversity and in consequence pollinator richness are necessary to reduce the impacts of wind and water stress.

Local loss of specific pollinators due to climate change can decrease the area of certain plants (Shi et al., 2021). Some pollinator‐dependent plants are important for erosion prevention, soil fertility, or soil biodiversity: The common pollinator‐dependent mangrove *Avicennia germinans* hosts arbuscular mycorrhizal fungi specialized on different salt concentrations (Vanegas et al., 2019). Pollinator‐loss‐induced depletion and final local extinction of *A. germinans* would affect these fungi and many other soil organisms living sheltered by the ramified root systems. Loss of these mangroves could also endanger more inland soil and its organisms depending on freshwater environment and not able to survive saltwater floods, which are currently balanced by pollinator‐dependent mangroves. *Cornus mas*, *Tilia cordata*, *Tilia platyphyllus*, *Salix caprea*, *Prunus spinosa*, and many other trees depend on pollinators to produce seed. Their root systems create a specific environment for arbuscular mycorrhizal fungi, the roots store carbon and prevent soil erosion. Pollinator‐dependent *Artemisia diffusa*, the prevailing rangeland shrub in Uzbekistan (Gintzburger, 2003), has a widely branched deep root system that supports water and carbon storage in soil, prevents erosion, and creates a specific environment for soil organisms. *A. diffusa* is the main wild forage resource for small ruminants in Central Asian rangelands (Gintzburger, 2003). Pollinator loss might lead to increase of unpalatable plants like *Peganum harmala*, in consequence abundance of dung beetles and soil fertility might decrease in such areas.

Many pollinator‐dependent plants contribute to soil fertility. For example, *Vicia faba* roots host nitrogen‐fixing bacteria, green manures like *Phacelia crenulate* and *Trifolium* enhance organic matter and thus support earthworms and soil fertility (https://www.rhs.org.uk/advice/profile?pid=373).

## CONCLUSION

Organizations supervising and directing conservation of soil biodiversity should protect ground‐nesting pollinators based on the habitat‐oriented CBD definition. Protection of ground‐nesting pollinators within soil biodiversity requires:

1. Pesticide risk assessments analyzing impacts on a mix of different solitary ground‐nesting pollinator species including impacts of delayed and combined effects under field conditions and contact exposure,
2. Knowledge‐raising campaigns concerning ground‐nesting pollinators,
3. Promotion of at least one dedicated area without second tillage, heavy machinery, and pesticides in each square kilometer of arable land. These are not necessarily fallow areas, as many ground‐nesting pollinators prefer arable land or pastures (Cope et al., 2019; Skidmore et al., 2019). Within the land‐sharing approach Farming with Alternative Pollinators (FAP; Christmann, Aw‐Hassan, et al., 2021; Christmann, Bencharki, et al., 2021; Christmann et al., 2017) this land can be used for (perennial) marketable habitat enhancement plants (MHEP), fruit trees, berries, cactus, alfalfa, mint, lavender, etc., planted as corridors or hedgerows. The insect diversity attracted by MHEP enhances the productivity of pollinator‐dependent main crops (Christmann, Aw‐Hassan, et al., 2021; Christmann, Bencharki, et al., 2021; Christmann et al., 2017).

When charging FAO with soil biodiversity and pollinator protection in 2002, CBD explicitly aimed at synergies, however in reality this has not happened and is overdue. CBD might wish to insist on correcting the current mechanistic FAO approach towards integrated protection of soil biodiversity that includes soil organisms such as ground‐nesting pollinators.

## CONFLICT OF INTEREST

The author declares no conflict of interest.
